# Regulation of gamma-secretase activating protein by the 5Lipoxygenase: *in vitro* and *in vivo* evidence

**DOI:** 10.1038/srep11086

**Published:** 2015-06-16

**Authors:** Jin Chu, Jian-Guo Li, Nicholas E. Hoffman, Alexandra M. Stough, Muniswamy Madesh, Domenico Praticò

**Affiliations:** 1 Department of Pharmacology, Center for Translational Medicine Temple University School of Medicine, Philadelphia, PA, USA; 2 Department of Biochemistry, Center for Translational Medicine Temple University School of Medicine, Philadelphia, PA, USA

## Abstract

The formation of Aβ is directly controlled by the γ-secretase complex and its activator, γ-secretase activating protein (GSAP). GSAP derives from a C-terminal fragment of a larger precursor protein via a caspase-3 mediated cleavage. However, the mechanism regulating this process remains unknown. Here we provide *in vitro* experimental evidence that 5-Lipoxygenase (5LO) is as an endogenous regulator for GSAP formation, but not for other known γ-secretase modulators, by directly and specifically activating caspase-3. These results were confirmed *in vivo* by using transgenic mouse models of Alzheimer’s disease in which 5LO level and activity were modulated genetically or pharmacologically. Taken together, our findings demonstrate that GSAP cleavage via caspase-3 is regulated and depend upon the availability of 5LO further establishing this protein as an attractive and viable therapeutic target for Alzheimer’s disease.

Alzheimer’s disease (AD) is the most prevalent cause of dementia and is associated with accumulation of amyloid-β peptide (Aβ) which is a major characteristic of the AD brain and responsible for some of its clinical manifestations^1^. While early-onset AD results from the mutations of genes that are involved in Aβ formation, a combination of environmental risk factors and different genes have been implicated in its sporadic form^2^. Among the latter, recent work has highlighted the potential role that the 5-Lipoxygenase (5LO) enzyme plays in AD pathogenesis by showing its involvement in Aβ formation and deposition^3^,^4^,^5^.

Activation of the γ-secretase complex is required for the final formation of Aβ peptides, and decreasing Aβ production by blocking this complex as a disease modifying approach for the treatment of AD has received intense investigation^6^. However, γ-secretase is known to process multiple substrates in addition to amyloid precursor protein (APP), most notably Notch, and this fact has severely limited the clinical development of inhibitors directly and irreversibly targeting this enzyme^7^. The recent discovery of a γ-secretase activating protein (GSAP) which interacts with this protease to facilitate Aβ formation without affecting Notch has established it as a relevant target for a viable and safer anti-Aβ therapy^8^,^9^. GSAP is increased in post-mortem brain tissues of AD patients, and its pharmacological or genetic inhibition results in an amelioration of the AD-like amyloidotic phenotype in transgenic mouse models of the disease^10^,^11^.

Recently, we identified a caspase-3 processing domain in the GSAP precursor protein sequence and provided experimental evidence that this caspase is involved in the formation of its active fragment, GSAP 16 kDa, and subsequent biogenesis of Aβ peptides^12^. Thus, while we are learning more about the neurobiology of GSAP, no information is available about the mechanism regulating its formation.

In the current paper, we provide experimental evidence that proteolytic formation of GSAP via caspase-3 is dependent upon the availability of the 5LO.

## Results

### *In vitro* studies

#### Modulation of GSAP formation by 5LO is specific

**Figure 1:**
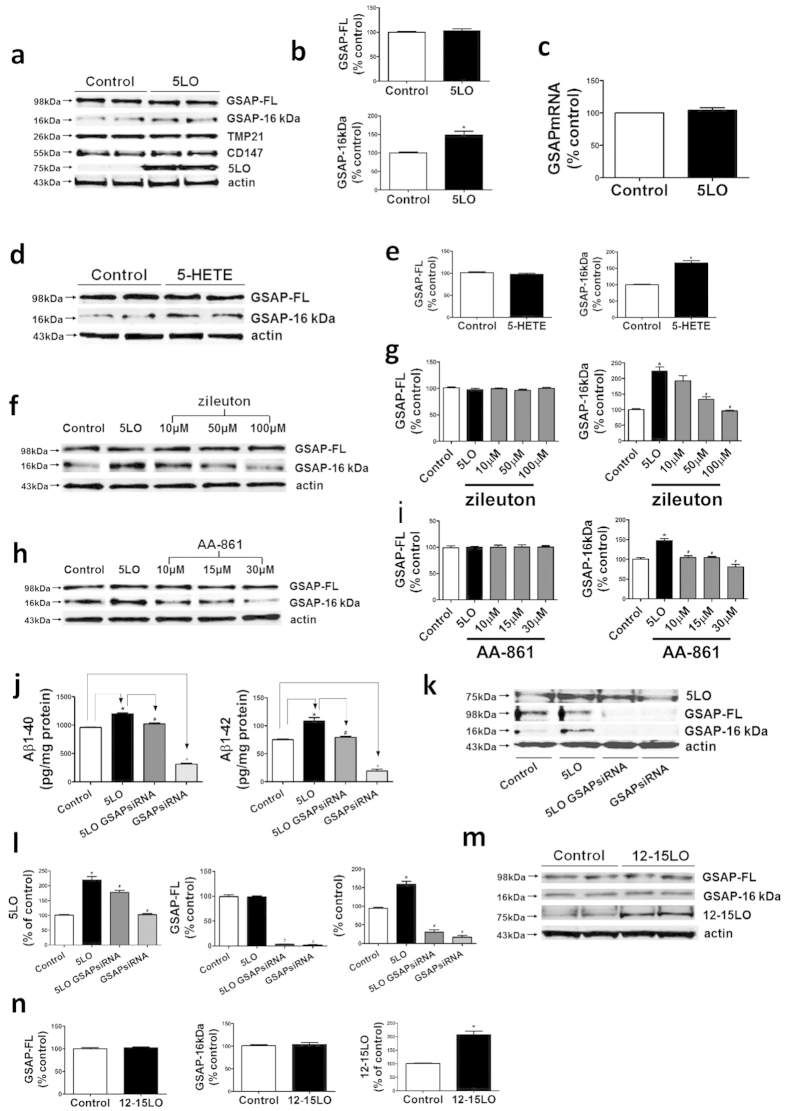
5LO specifically regulates GSAP 16kDa formation. N2A-APPswe cells were transfected with 5LO cDNA then treated with Zileuton and AA-861 overnight separately; cells also were treated with 5-HETE or vehicle alone, supernatant and cell lysates collected for analysis. (**a**) Representative western blot analysis of full length GSAP (GSAP-FL), GSAP-16 kDa, TMP21, CD147, and 5LO in lysates from cells treated with empty vector (control) or 5LO cDNA (5LO). (**b**) Densitometric analyses of the immunoreactivities to the antibodies shown in panel a (*p = 0.01) (n = 3). (**c**) GSAP mRNA levels in N2A-APPswe cells transfected with 5LO cDNA (5LO) or empty vector (control). (**d**) Representative western blot analysis of GSAP-FL and GSAP-16 kDa in cells treated with 5-HETE (10 μM) or vehicle (control). (**e**) Densitometric analyses of the immunoreactivities to the antibodies shown in panel d (*p = 0.0006) (n = 3). (**f,h**) Representative western blot analysis of GSAP-FL and GSAP-16 kDa in cells transfected with 5LO cDNA and then treated with Zileuton or AA-861. (**g,i**) Densitometric analyses of the immunoreactivities to the antibodies shown in panel f and h, respectively (*p = 0.001; ^#^p = 0.005; ^#^p = 0.0009) (*p = 0.002; ^#^p = 0.004; ^#^p = 0.002; ^#^p = 0.001) (n = 3). (**j)**. Levels of Aβ 1-40 and Aβ 1-42 in conditioned media from cells transfected with empty vector (control), 5LO cDNA, GSAP siRNA and 5LO, and GSAP siRNA alone (*p < 0.0001; ^#^p < 0.0001; ^^^p < 0.0001) (*p = 0.007; p = 0.01; ^p < 0.0001) (n = 3). Results are mean ± s.e.m. (**k**) Representative western blot analysis of GSAP-FL, GSAP-16 kDa, and 5LO in the lysates from the same cells. (**l**) Densitometric analyses of the immunoreactivities to the antibodies shown in panel k (*p = 0.0005, ^#^p = 0.03; ^#^p < 0.0001, ^#^p < 0.0001; ^#^p = 0.001, ^#^p = 0.0002, ^#^p < 0.0001) (n = 3). (**m**) Representative western blot analysis of GSAP^-^FL and GSAP-16 kDa in the lysates of cells transfected with 12-15LO cDNA (12-15LO) or empty vector (control). (**n**) Densitometric analyses of the immunoreactivities to the antibodies shown in panel m (*p = 0.002) (n = 3).

Compared with empty vector, neuronal N2A-APPswe cells over-expressing 5LO had a significant increase in GSAP-16 kDa fragment levels but not its precursor protein, GSAP-FL (Fig. 1a–b). By contrast, no significant changes were detected for two other γ-secretase modulatory proteins: TMP21 and CD147 (Fig. 1a)^13^,^14^. Under this experimental condition, quantitative real time PCR did not show any significant difference in GSAP mRNA levels between the two groups (Fig. 1c). In the same cells addition of the major 5LO metabolite, 5-HETE, resulted in a significant increase of GSAP-16 kDa levels (Fig. 1d,e), whereas two selective 5LO pharmacological inhibitors, zileuton and AA-861, lowered GSAP-16 kDa levels in a dose-dependent manner (Fig. 1f–i)^15^. The Aβ 1–40 and 1–42 levels in supernatants from cells over-expressing 5LO were significantly increased, however, when the cells were treated with GSAP siRNA these levels were decreased (Fig. 1j), as were both the GSAP-FL and its 16 kDa fragment (Fig. 1k,l). In contrast, no significant changes for both GSAP-FL and the 16 kDa fragment levels were detected when neuronal cells were over-expressing another LO, the 12–15LO (Fig. 1m,n).

**Figure 2:**
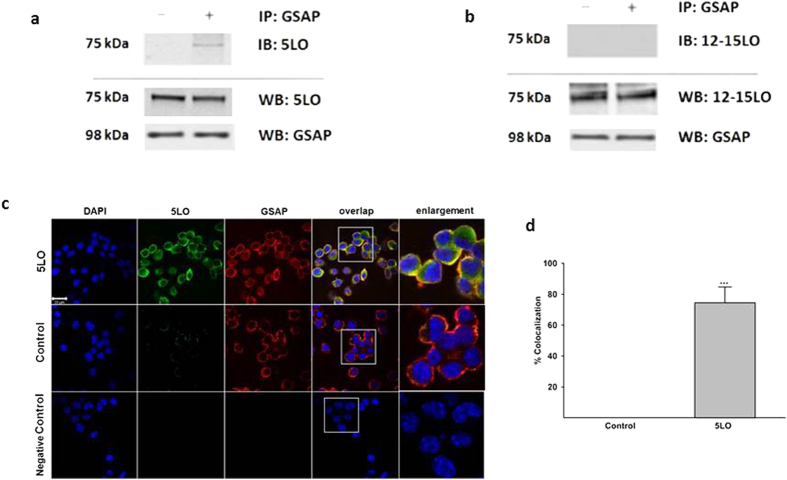
5LO interacts and co-localizes with GSAP. N2A-APPswe cells were transfected with 5LO cDNA, cell lysates collected for immunoprecipitation analysis. (**a**) Representative western blot analysis showing that GSAP co-immunoprecipitates with 5LO. Samples were immunoprecipitated (IP) with an antibody against GSAP, then immunoblotted (IB) with an antibody against 5LO. As control, total protein lysates were immunoblotted by Western blot by using an antibody against 5LO and GSAP. (**b**) Samples were immunoprecipitated (IP) with an antibody against GSAP, then immunoblotted (IB) with an antibody against 12-15LO. As control, total protein lysates were immunoblotted by Western blot by using a 12-15LO and GSAP antibodies. (**c**) Representative images of immunofluorescence analysis of cells transfected with 5LO cDNA and incubated with primary antibody for 5LO (green), and GSAP (red) (scale bar: 20 μm). (**d**) Intensity histogram shows the colocalization percentage of 5LO (green channel) and GSAP (red channel).

Co-immunoprecipation studies revealed the physical interaction between GSAP and 5LO, but not for GSAP and 12–15LO (Fig. 2a,b). This observation was further substantiated by immunofluorescence studies showing a significant cellular co-localization between GSAP and 5LO (Fig. 2c,d).

#### Caspase-3 is required for 5LO-dependent GSAP formation

**Figure 3:**
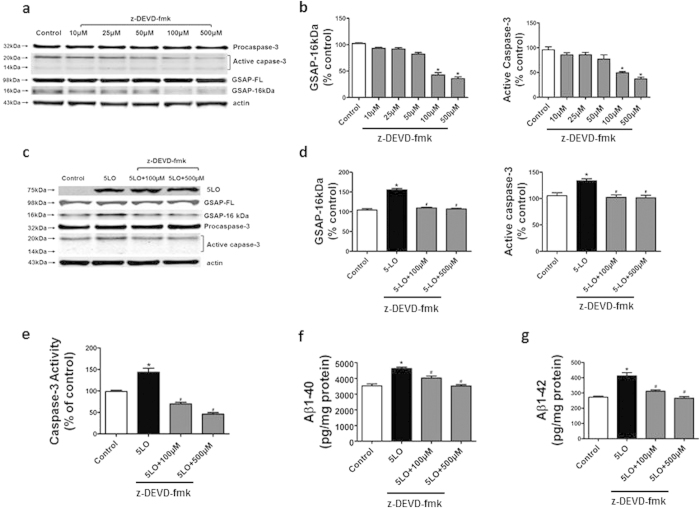
Pharmacological blockade of caspase-3 prevents 5LO-dependent GSAP formation. N2A-APPswe cells were treated with z-DEVD-fmk alone overnight, or transfected with 5LO cDNA and then treated with increasing concentration of the same selective caspase-3 inhibitor (z-DEVD-fmk). Cell lysates were collected for analysis. (**a**) Representative western blot analysis of full length GSAP (GSAP-FL), GSAP-16 kDa, procaspase-3, and active caspase-3 in lysates from cells incubated with increasing concentration of z-DEVD-fmk or vehicle (control). (**b**) Densitometric analyses of the immunoreactivities to GSAP-16 kDa and active caspase-3 shown in panel a (*p = 0.0003). (**c**) Representative western blot analysis of GSAP-FL, GSAP-16 kDa, procaspase-3, and active caspase-3 in the lysates of cells transfected with empty vector (control), 5LO cDNA, or 5LO cDNA in the presence of z-DEVD- fmk (100 and 500 μM) (**d**) Densitometric analyses of the immunoreactivities to GSAP-16 kDa and active caspase-3 shown in panel c (*p = 0.0005; ^#^p ≤ 0.0004). **e**. Caspase-3 activity measured in the lysates of cells transfected with empty vector (control), 5LO cDNA, or 5LO and z-DEVD- fmk (*p = 0.0005; ^#^p ≤ 0.007). (**f**) Levels of Aβ 1–40 in conditioned media from the same cells (*p = 0.0004; ^#^p ≤ 0.009). Values represent mean ± s.e.m. (**g**) Levels of Aβ 1–42 in conditioned media from the same cells (*p = 0.0007; ^#^p = 0.0004; ^#^p = 0.0008). Values represent mean ± s.e.m.

Having shown that 5LO specifically modulate the formation of GSAP16 kDa, and with the knowledge that GSAP precursor is cleaved by caspase-3^12^, next we investigated whether 5LO action on GSAP was dependent or independent of this very caspase. First, neuronal cells were incubated with increasing doses of a specific and cell permeable caspase-3 inhibitor, z-DEVD-fmk^16^, and the effect on GSAP 16 kDa assessed. As shown in Fig. 3, we observed that pharmacological inhibition of caspase-3 activation resulted in a significant decrease in the active form of caspase-3 and GSAP 16 kDa levels, but no changes were detected for procaspase-3 and GSAP-FL levels (Fig. 3a,b). Incubation of the same inhibitor with 5LO overexpressing cells resulted in a significant reduction in GSAP 16 kDa, active caspase-3 levels and activity, and Aβ levels, but no changes were observed for GSAP-FL and procaspase-3 protein levels (Fig. 3c–g).

**Figure 4:**
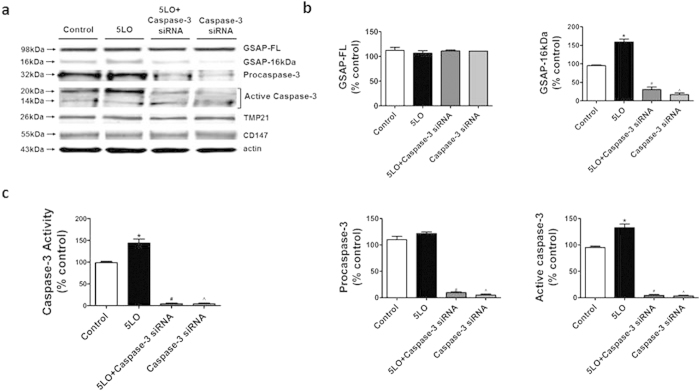
Gene silencing of caspase-3 prevents 5LO-dependent formation of GSAP. N2A-APPswe cells were transfected with 5LO cDNA overnight alone or in the presence of caspase-3 siRNA for 48hrs, then cell lysates collected for analysis. (**a**) Representative Western blot analysis of full length GSAP (GSAP-FL), GSAP-16kDa, TMP21 and CD147, procaspase-3, and active caspase-3 in lysates from cells transfected with empty vector (control), 5LO cDNA, caspase-3 siRNA (100 nM) and 5LO cDNA, caspase-3 siRNA alone.(**b**) Densitometric analyses of the immunoreactivities shown in panel a for GSAP-FL, GSAP 16 kDa (*p = 0.001; ^#^p = 0.0001; ^^^p = 0.0001), pro-casapse-3 (^#^p < 0.0001; ^^^p < 0.0001), and active caspase-3 (*p = 0.008; ^#^p < 0.0001; ^^^p < 0.0001). (**c**) Caspase-3 activity measured in in lysates from the same cells described in panel a (*p = 0.004; ^#^p < 0.0001; ^^^p < 0.0001). Values represent mean ± s.e.m.

To further confirm this finding, next we used caspase-3 siRNA to knock-down this protease and evaluated the effect on GSAP fragment formation in cells over-expressing 5LO. As shown in Fig. 4, cells treated with this siRNA showed a dramatic reduction in pro-caspase-3, active caspase-3 steady state levels, which was accompanied by a decrease of GSAP-16 kDa (Fig. 4a,b). By contrast, no differences in GSAP-FL, TMP21 and CD147 were detected between 5LO overexpressing cells treated or not treated with caspase-3 siRNA (Fig. 4a,b). Under these experimental conditions, we found that *in vitro* caspase-3 activity levels were significantly reduced (Fig. 4c).

### *In vivo* studies

#### 5LO modulates GSAP formation via caspase-3

Next, we wanted to confirm these findings *in vivo* by using brain tissues from 3xTg mice over-expressing 5LO (3xTg/H5LO), 3xTg genetically deficient for 5LO mice (3xTg 5LOKO), and 3xTg mice treated with zileuton, a selective 5LO inhibitor (3xTg zileuton), all of which have been previously described^5^,^17^,^18^.

**Figure 5:**
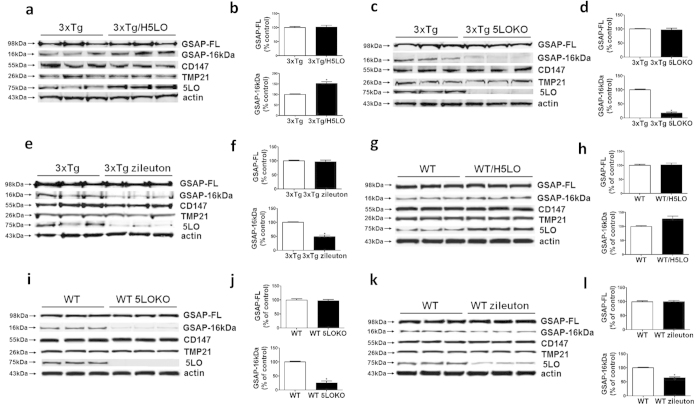
Genetic or pharmacological manipulation of 5LO modulates GSAP 16 kDa levels in mouse brains. (**a**) Representative Western blot analysis of full length GSAP (GSAP-FL), GSAP-16 kDa, TMP21, CD147, and 5LO in brain homogenates from control mice (3xTg) or mice over-expressing 5LO (3xTg/H5LO). (**b**) Densitometric analyses of the immunoreactivities to GSAP-FL and GSAP-16 kDa shown in panel a (n = 3 per group, *p = 0.007). (**c**) Representative Western blot analysis of GSAP-FL, GSAP-16 kDa, TMP21, CD147, and 5LO in brain homogenates from 3xTg mice or 3xTg mice genetically deficient for 5LO (3xTg 5LOKO). (**d**) Densitometric analyses of the immunoreactivities to GSAP-FL and GSAP-16kDa shown in panel c (n = 3 per group, *p < 0.0001). (**e**) Representative western blots of GSAP-FL, GSAP-16 kDa, TMP21, CD147, and 5LO in the cortex of 3xTg mice receiving zileuton or placebo (3xTg). (**f**) Densitom**e**tric analyses of the immunoreactivities to GSAP-FL and GSAP-16 kDa shown in panel e (n = 3 per group, *p = 0.001). Values represent mean ± s.e.m. (**g**) Representative Western blot analysis of full length GSAP (GSAP-FL), GSAP-16 kDa, TMP21, CD147, and 5LO in brain homogenates from control mice (WT) or mice over-expressing 5LO (WT/H5LO). (**h**) Densitometric analyses of the immunoreactivities to GSAP-FL and GSAP-16 kDa shown in panel g (n = 3 per group). (**i**) Representative Western blot analysis of GSAP-FL, GSAP-16kDa, TMP21, CD147, and 5LO in brain homogenates from WT mice or WT mice genetically deficient for 5LO (WT 5LOKO). (**j**) Densitometric analyses of the immunoreactivities to GSAP-FL and GSAP-16 kDa shown in panel i (n = 3 per group, *p = 0.0005). (**k**) Representative western blots of GSAP-FL, GSAP-16 kDa, TMP21, CD147, and 5LO in the cortex of WT mice receiving zileuton or placebo (WT). (**l**) Densitometric analyses of the immunoreactivities to GSAP-FL and GSAP-16 kDa shown the panel k (n = 3 per group, *p = 0.001). Values represent mean ± s.e.m.

Compared with controls, 3xTg/H5LO mice had a significant increase in GSAP-16 kDa fragment levels, whereas no significant differences were observed between the two groups for GSAP-FL, TMP21 and CD147 steady state levels (Fig. 5a,b). By contrast, we observed that 3xTg mice genetically deficient for 5LO, or receiving the 5LO inhibitor had significant decreases in GSAP-16 kDa fragment levels but no changes in GSAP-FL, TMP21 and CD147 levels when compared to their control groups (Fig. 5c–f). Next, we also tested the effect of the 5LO modulation on GSAP fragment expression in wild-type mice. Compared with controls, WT/H5LO mice had an increase in GSAP-16 kDa fragment levels, which did not reach statistical significance, whereas no significant differences were observed between the two groups for GSAP-FL, TMP21 and CD147 steady state levels (Fig. 5g,h). By contrast, we observed that WT mice genetically deficient for 5LO, or receiving the 5LO inhibitor had significant decreases in GSAP-16 kDa fragment levels but no changes in GSAP-FL, TMP21 and CD147 levels when compared to their control groups (Fig. 5i–l).

**Figure 6:**
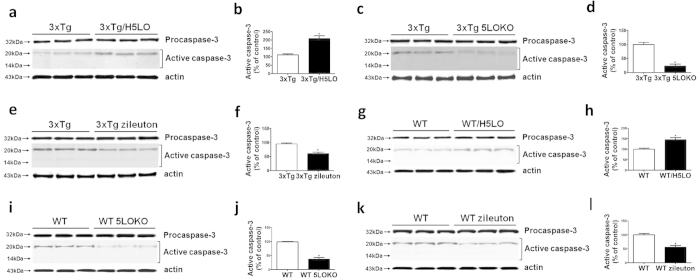
(Expression levels of 5LO modulates active caspase-3 in mouse brains. (**a**) Representative Western blot analysis of procaspase-3 and active caspase-3 in brain homogenates from control mice (3xTg), or mice over-expressing 5LO 3xTg/H5LO). (**b**) Densitometric analyses of the immunoreactivities to caspase-3 shown in panel a (n = 3 per group, *p = 0.004). (**c**) Representative Western blot analysis of procaspase-3 and active caspase-3 in brain homogenates from 3xTg mice, or 3xTg mice genetically deficient for 5LO (3xTg 5LOKO). (**d**) Densitometric analyses of the immunoreactivities to active caspase-3 shown in panel c (n = 3 per group, *p = 0.002). (**e**) Representative western blots of procaspase-3 and active caspase-3 in the cortex of 3xTg mice receiving zileuton or placebo for 10 months. (**f**) Densitometric analyses of the immunoreactivities to active caspase-3 shown in the panel e. (n = 3 per group, *p = 0.005). Values represent mean ± s.e.m. (**g**) Representative Western blot analysis of procaspase-3 and active caspase-3 in brain homogenates from control mice (WT), or mice over-expressing 5LO (WT**/**H5LO). (**h**) Densitometric analyses of the immunoreactivities to caspase-3 shown in panel g (n = 3 per group, *p = 0.009). (**i**) Representative Western blot analysis of procaspase-3 and active caspase-3 in brain homogenates from WT mice, or WT mice genetically deficient for 5LO (WT 5LOKO). (**j**) Densitometric analyses of the immunoreactivities to active caspase-3 shown in panel i (n = 3 per group, *p = 0.0009). (**k**) Representative western blots of procaspase-3 and active caspase-3 in the cortex of WT mice receiving zileuton or placebo for 10 months. (**l**) Densitometric analyses of the immunoreactivities to active caspase-3 shown in panel k.(n = 3 per group, *p = 0.007).

In addition, we found a significant increase in active caspase-3 but no change in procaspase-3 levels in 3xTg mice over-expressing 5LO compared to the control group (Fig. 6a,b). The opposite was true for both 3xTg mice genetically deficient for 5LO and receiving 5LO inhibitor zileuton (Fig. 6c–f). Similar results were observed in brains from WT mice (Fig. 6g–l).

**Figure 7:**
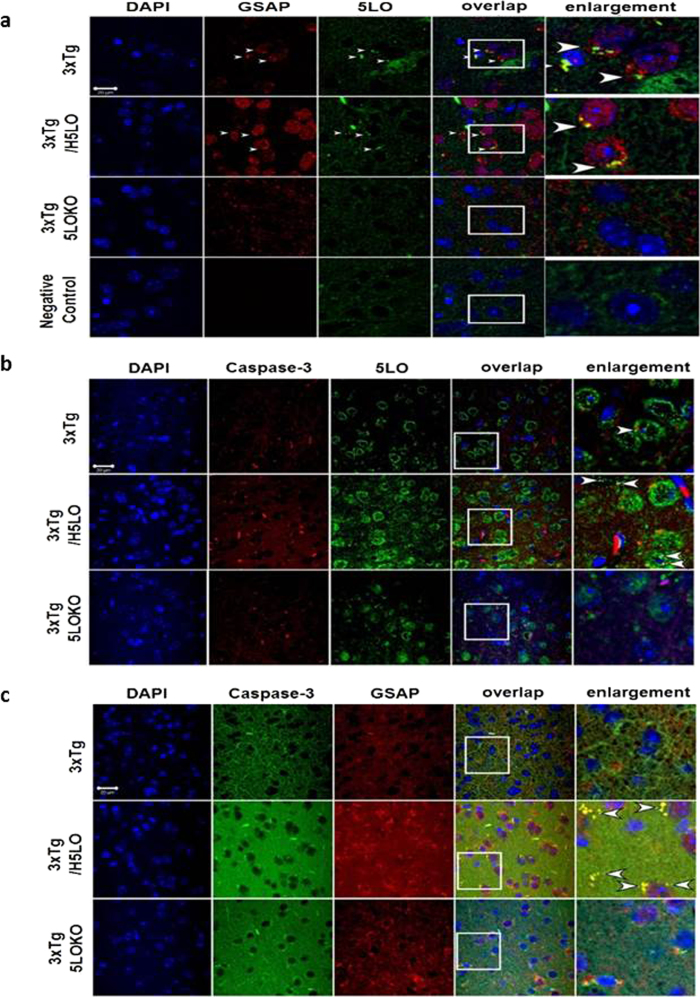
Co-localization of 5LO with GSAP in brain of 3xTg mice. Representative images of immunofluorescence analysis of brain sections of control mice (3xTg), mice over-expressing 5LO (3xTg/H5LO), and mice genetically deficient for 5LO (3xTg 5LOKO). Panel a sections were incubated with primary antibody for 5LO (green), and GSAP (red); panel b, sections were incubated with primary antibody for 5LO (green), and caspase-3 (red); panel c sections were incubated with primary antibody for caspase-3 (green), and GSAP (red). Negative control panels show images of brain sections incubated only with the secondary antibody. Scale bar: 20 μm.

Finally, immunofluorescence studies showed that compared with 3xTg control mice the co-localization for GSAP and 5LO, caspase-3 and 5LO, and caspase-3 and GSAP were higher in 3xTg mice overexpressing 5LO, whereas this was absent in 3xTg mice genetically deficient for 5LO (Fig. 7a–c).

## Discussion

In the current paper we provide *in vitro* and *in vivo* experimental evidence that 5LO is involved in the proteolytic processing of the GSAP-FL, the precursor protein that by generating the biologically active fragment GSAP 16 kDa is ultimately responsible for controlling Aβ biogenesis, and that this biological effect is mediated by the activation of caspase-3. In addition, our findings demonstrate that this process is selective for GSAP, since it does not influence other γ-secretase modulators, and specific for 5LO since another LO, the 12/15LO, is without effect.

The 5LO is an enzyme widely expressed in the central nervous system and is up-regulated in AD brains^19^. Consistent evidence has demonstrated that this protein is directly involved in the production and subsequent deposition of Aβ peptides *in vitro* and *in vivo*^20^. GSAP is a key molecule responsible for the rate-limiting step in Aβ production by directly interacting with the γ-secretase complex, and for this reason it is an emerging potential new player in molecular and cellular mechanisms of relevance to AD brain amyloidosis^21^,^22^.

Because of these two pieces of information, in the current paper we wanted to test the hypothesis that 5LO act as a direct regulator of GSAP formation, and if this was the case, investigate the underlying mechanism(s).

Initially, we demonstrated that over-expression of 5LO, but not 12/15LO, did not alter steady state and mRNA levels of the GSAP precursor protein, but selectively increased the amount of its active form, the GSAP16 kDa. To further investigate the specificity of this biological effect we looked at 2 different γ-secretase protein modulators, namely TMP21 and CD147, and we observed that 5LO was without any effect. These data were affirmed in the inverse by using pharmacological and genetic strategies. Thus, incubation with two distinct and structurally unrelated 5LO inhibitors or the use of GSAP siRNA reduced GSAP 16 kDa levels. Next, by using biochemistry and immunofluorescence assay approaches we showed that in neuronal cells, GSAP physically and directly interacts with 5LO but not with another member of the LO family (i.e,12/15LO).

Because our previous study identified a caspase-3 processing domain in the GSAP-FL precursor protein sequence and provided experimental evidence that this caspase is essential for GSAP 16 kDa formation and biogenesis of Aβ peptides^12^, we wanted to investigate whether the 5LO effect above described was also mediated by a caspase-3 dependent mechanism.

Interestingly, this hypothesis was supported by some previous data in the literature suggesting a biological link between 5LO and caspase-3. Thus, ApoEˉ/ˉ mice lacking 5LO have reduced caspase-3 activity^23^, while overexpression of 5LO significantly enhanced caspase-3 activation in PC12 cells^24^, and 5LO pharmacological inhibition significantly down-regulated caspase-3 activation in the brains of rats undergoing middle cerebral artery occlusion^25^.

To prove that 5LO regulation of GSAP 16 kDa formation is dependent upon caspase-3 activation, we adopted a pharmacological and a genetic approach.

First, we showed that 5LO overexpression was not able to influence GSAP precursor protein processing to form the 16 kDa fragment in the presence of a caspase-3 inhibitor. Under this experimental condition we observed a significant reduction in the activity of this caspase and in the amount of Aβ in the supernatants. Similar results were obtained when levels of procaspase-3 were significantly reduced in neuronal cells treated with caspase-3 siRNA.

Taken together our findings provide *in vitro* experimental support for the novel hypothesis that 5LO specifically regulates the processing of GSAP-FL and amyloidogenesis via the activation of caspase-3. This observation does not contrast with our previous report in which we showed that 5LO can act as a modulator of Aβ formation by regulating s the transcription of the four components of the γ-secretase complex^9^. On the other hand, we believe that this newly discovered function of 5LO add a new interesting facet to the complex neurobiology of this protein within the central nervous system.

The biological relevance of our discovery was corroborated by the implementation of ex-vivo studies in a transgenic mouse model of AD in which the 5LO pathway was genetically or pharmacologically modulated.

Thus, genetic absence or pharmacological inhibition of 5LO resulted in a significant reduction in GSAP 16 kDa, but no changes in its precursor protein, TMP21 nor CD147 levels in the brains of 3xTg mice. By contrast, brain tissues from 3xTg mice over-expressing 5LO had a significant increase in GSAP16 kDa, but no changes in GSAP-FL, TMP21 and CD147 steady state levels. These results were reproduced in WT mice, suggesting that 5LO acts as an endogenous modulator of GSAP fragment expression independently from the presence of a transgene.

Importantly, the amount or availability of 5LO was a direct predictor of the levels of active caspase-3 in the brains of these animals, where immunofluorescence studies revealed an increase or decrease in co-localization between GSAP and 5LO, caspase-3 and 5LO, and caspase-3 and GSAP, respectively.

In summary, our studies provide the first direct experimental evidence that GSAP cleavage via caspase-3, which generates the 16 kDa fragment ultimately responsible for controlling the γ-secretase activity and amyloidogenesis *in vivo*, is specifically regulated and dependent upon the availability of 5LO.

They further establish 5LO as an attractive and viable Aβ lowering therapeutic target for AD without the toxic effect of classic γ-secretase inhibitors.

## Methods

### Cell culture and treatments

The N2A (neuro-2 A neuroblastoma) neuronal cells stably expressing human APP carrying the K670 N, M671 L Swedish mutation (APP swe) were used in our *in vitro* studies and grown, as previously described^12^,^26^. For transfection, cells were grown to 70% confluence and transfected with 1 μg of empty vector (pcDNA3.1) or human 5LO pcDNA3.1 (Dr. Colin Funk, Queen’s University, Kingston, Canada) by using Lipofectamine 2000® (Invitrogen, Carlsbad, CA) according to the manufacturer’s instructions. After 24 h transfection, supernatants were collected, and cells pellets harvested in lytic buffer for biochemical analyses. For pharmacological treatment, cells were incubated with 5-HETE (10 μM), or zileuton (10 μM, 50 μM, 100 μM), or AA-861 (10 μM, 15 μM, 30 μM), or with the caspase-3 inhibitor (z-DEVD-fmk) (100 μM, 500 μM) for 24 h after transient transfection with human 5LO pcDNA3.1, or singly incubated with the caspase-3 inhibitor (z-DEVD-fmk) (10 μM, 25 μM, 50 μM, 100 μM, 500 μM) for 24 hours, after which supernatants were collected, and cells pellets harvested in lytic buffer for biochemical analyses. For siRNA knock-down studies, GSAP siRNA (sc-140659), and caspase-3 siRNA (sc-29927), and a negative control siRNAs (Control siRNA-A, sc-37007) were all obtained from Santa Cruz Biotech. N2Asw-APP cells were reverse transfected with siRNA (100 nM) using Lipofectamine® 2000 Transfection Reagent (Invitrogen, Carslab, CA) according to the manufacturer’s instruction and as previously described^12^,^26^.

### Mice and treatments

All animal procedures were approved by the Animal Care and Usage Committee of Temple University, in accordance with the U.S. National Institutes of Health guidelines. The 3xTg mice, 3xTg/H5LO mice over-expressing 5LO (3xTg/H5LO), 3xTg mice genetically deficient for 5LO (3xTg/5LOKO), the 3xTg mice receiving zileuton, and their wild type control mice were described previously^5^,^17^,^18^. At sacrifice, brains were removed and dissected in two hemihalves by mid-sagittal dissection. One half was immediately stored at 80 ^o^C for biochemistry assays, the other immediately fixed for immunofluorescence studies.

### Quantitative analysis of Aβ peptides

Levels of Aβ 1–40 and 1–42 in supernatants were assayed by a sensitive sandwich ELISA kit (WAKO Chem., Richmond, VA), as previously described^17^,^18^.

### Immunoblot analysis

Proteins were extracted, sonicated, centrifuged at 13,000 rpm for 45 min at 4 ^o^C, and supernatants used for immunoblot analysis, as previously described^17^,^18^. Actin was always used as an internal loading control. Primary antibodies used were as follows: anti GSAP full length (1:200) and GSAP-16 kDa (1:150) (Thermo Scientific); anti-5LO (1:500) (BD Bioscience); anti-12-15LO (1:200) (Santa Cruz Biotech., Dallas, TX); anti-TMP21 (1:200), anti-CD147(1:200), anti-caspase-3 (1:200) (Santa Cruz Biotech., Dallas, TX); anti-β-actin (1:200) (Santa Cruz Biotech., Dallas, TX). IRDye infrared secondary antibodies were from LI-COR Bioscience (Lincoln, NE).

### Co-immunoprecipitation studies

Cells were grown to 85–90% confluence and then lysed in 50 mM HEPES, 150 mM NaCl, 5 mM MgCl_2_, 5 mM CaCl_2_, 1% CHAPSO containing a protease inhibitor mixture. Prior to immunoprecipitation, cell lysates were diluted in lysis buffer lacking CHAPSO to give 0.25% final CHAPSO concentration. Cell lysates were incubated for 3 hr at room temperature with 5 μg of anti-GSAP antibody. DynabeadsM-280 sheep anti-rabbit IgG (50 μl; Invitrogen, Carslab, CA) were added and samples were incubated overnight at 4 °C. A control incubation of cell lysates with Dynabeads alone was also conducted. Dynabeads were collected and washed 5 times with lysis buffer containing 0.25% CHAPSO. Bound proteins were eluted with SDS sample buffer containing reducing agent and subject to Western blot analysis as described in the previous paragraph.

### Immunofluorescence microscopy

Immunofluorescence studies were performed as previously described^12^. Briefly, cells were placed on glass coverslips and the following day fixed in 4% paraformaldehyde in PBS for 15 min at 22 °C. For the brain tissues, sections were deparaffinized, hydrated and subsequently with 3% H_2_O_2_ in methanol, and then retrieved antigen with citrate (10 mM). After five rinses with PBS, cells or sections were incubated in a blocking solution (5% normal serum/0.4% TX-100) for 1 h at 22 °C and then with the primary antibody separately against GSAP, 5LO, caspase-3 overnight at 4 °C. After washing with PBS, samples were incubated for 1 h with a secondary Texas Red or Alexa Fluor 488- conjugated antibody (Invitrogen, Carslab, CA). Coverslips were mounted using VECTASHIELD mounting medium (Vector Laboratories, Burlingame, CA, USA) and analyzed with Confocal Laser Scanning Microscope Carl Zeiss LSM 710 (Carl Zeiss, Germany). Control coverslips were processed as described above except that no primary antibody was added to the solution.

### Caspase-3 activity assay

Cells were rinsed with PBS once and lysed in buffer A (50 mM Tris–HCl [pH 8.0], 150 Mm sodium chloride, 1% NP-40, 0.5% sodium deoxycholate, 0.1% sodium dodecyl sulfate, 0.02% sodium azide and freshly added protease inhibitors [100 μg/ml phenylmethysulfonyl fluoride and 1 μg/ml Aprotinin]). Following incubation on ice for 0.5 h, the samples were centrifuged at 16,000 g at 4 °C for 15 min and the supernatant was collected. The caspase-3 activity was measured by using a colorimetric substrate Ac-DEVDpNA and the production of pNA was monitored over 20 min by microplate reader at OD405. One unit activity was defined as the amount of the enzyme required to cleave 1 pmol of pNA/min/mg protein. The activity of caspase-3 was also measured indirectly by cleavage of PARP, which is a recognized substrate of caspase-3.

### Real-Time Quantitative Reverse Transcription-PCR Amplification

RNA was extracted and purified using the RNeasy Mini Kit (Qiagen, Valencia, CA), as previously described^4^. Briefly, mouse GSAP gene was amplified by using the corresponding primers (SA Biosciences, Valencia, CA), and β-actin was always used as an internal control gene.

### Data analysis

One-way ANOVA followed by the Bonferroni’s Multiple Comparison tests and non-parametric t-tests were performed using GraphPad Prism 5.0. All data are presented as mean ± s.e.m. Significance was set at p < 0.05.

## Additional Information

**How to cite this article**: Chu, J. *et al.* Regulation of gamma-secretase activating protein by the 5Lipoxygenase: *in vitro* and *in vivo* evidence. *Sci. Rep.* **5**, 11086; doi: 10.1038/srep11086 (2015).
